# Adaptation of lodgepole pine and interior spruce to climate: implications for reforestation in a warming world

**DOI:** 10.1111/eva.12345

**Published:** 2016-01-19

**Authors:** Katharina J. Liepe, Andreas Hamann, Pia Smets, Connor R. Fitzpatrick, Sally N. Aitken

**Affiliations:** ^1^ Department of Renewable ResourcesUniversity of AlbertaEdmontonABCanada; ^2^ Department of Forest and Conservation SciencesUniversity of British ColumbiaVancouverBCCanada

**Keywords:** assisted migration, climate change, ecological genetics, genetic diversity, *Q*_ST_, seed zones

## Abstract

We investigated adaptation to climate in populations of two widespread tree species across a range of contrasting environments in western Canada. In a series of common garden experiments, bud phenology, cold hardiness, and seedling growth traits were assessed for 254 populations in the interior spruce complex (*Picea glauca, P. engelmannii,* and their hybrids) and for 281 populations of lodgepole pine (*Pinus contorta*). Complex multitrait adaptations to different ecological regions such as boreal, montane, coastal, and arid environments accounted for 15–20% of the total variance. This population differentiation could be directly linked to climate variables through multivariate regression tree analysis. Our results suggest that adaptation to climate does not always correspond linearly to temperature gradients. For example, opposite trait values (e.g., early versus late budbreak) may be found in response to apparently similar cold environments (e.g., boreal and montane). Climate change adaptation strategies may therefore not always be possible through a simple shift of seed sources along environmental gradients. For the two species in this study, we identified a relatively small number of uniquely adapted populations (11 for interior spruce and nine for lodgepole pine) that may be used to manage adaptive variation under current and expected future climates.

## Introduction

Temperate and boreal forest ecosystems of western Canada provide more than 60% of Canada's harvest volume, with lodgepole pine (*Pinus contorta* Dougl. ex Loud.) and interior spruce (*Picea glauca* [Moench] Voss, *P. engelmannii* Parry ex Engelm. and their hybrids) being the most important forestry species (Canadian Forest Service 2013). Climate change threatens these ecosystems and their economic benefits through direct impacts or insect and disease‐mediated damage (Allen et al. 2010; Michaelian et al. 2011; Peng et al. 2011). Potential adaptation strategies to address these problems include prescriptions that enhance the resistance and resilience of forest ecosystems (Millar et al. 2007) or human assisted migration of species, which has been proposed as key forest management strategy to maintain forest health and productivity (Wang et al. 2006; Pedlar et al. 2012). Assisted migration could also be restricted to assisted gene flow, the movement of populations within a species range (Gray et al. 2011; Aitken and Whitlock 2013).

Assisted gene flow of planting stock representing locally adapted populations requires knowledge of ecological genetics and extent of local adaptation of species (e.g., Morgenstern 1996; Alberto et al. 2013). This knowledge is integrated into reforestation prescriptions that restrict movement of seed through a system of seed zones and seed transfer guidelines (Ying and Yanchuk 2006). Local adaptation of forest trees has traditionally been revealed by reciprocal transplant experiments, also referred to as provenance trials. Forest geneticists have established and maintained large and systematic provenance trials since the 1950s, with the goal of identifying superior genotypes for reforestation. These trials have emerged as useful climate change laboratories, as first noted by Mátyás (1994). By testing populations collected throughout a range of source climates and grown across a range of recipient climates, provenance tests can predict the effects of climatic maladaptation under realistic forest plantation settings (Rehfeldt et al. 1999).

As per definition, ‘populations are locally adapted when populations have the highest fitness at their home sites and lower fitness in other parts of the range’ (Savolainen et al. 2007). One way to infer local adaptation is to use growth and survival data in provenance trial series over multiple environments as proxy for fitness. Genotype by environment interactions, with local populations performing best at their home sites, can be interpreted as strong support for optimal adaptation to local environments. However, the nature of such adaptations remains unknown without measuring adaptive traits and their associations with environmental variables directly. Genecology research reveals the underlying mechanisms of local adaptation by studying population differentiation in adaptive traits. For a strong inference of population differentiation representing adaptation, population differentiation needs to be interpreted in light of the source environments (e.g., frost hardy genotypes originating from cold environments). That said, genecology studies cannot prove that a particular trait value observed in a local population is optimal. Data of adaptive trait variation should therefore ideally be interpreted in combination with data from long‐term field testing to confirm local optimality.

Traits relevant for adaptation to climate include the phenology of budbreak and growth cessation, responsible for the synchronization of the growing period with the available growing season. Typically, chilling and heat sum requirements control budbreak (Hannerz 1999), while photoperiod sensing mechanisms control budset (e.g., Ekberg et al. 1979). Sometimes, phenology is modified by moisture availability to advance (White et al. 1979) or delay (Li et al. 2010) the onset of the growing period. The timing of spring and fall phenology is usually a trade‐off between maximizing use of the available growing season and avoiding cold damage from rare late frosts in spring or early frosts in fall (Leinonen and Hanninen 2002). Similarly, the onset and degree of cold hardiness in living tissue and wood properties are important adaptations to winter severity and length that require the plant to invest some resources into safety mechanisms at the expense of growth capacity (Howe et al. 2003; Schreiber et al. 2013).

To develop assisted gene flow prescriptions to address future climate change, it is necessary to ensure that multitrait adaptations of plant populations are matched with corresponding multivariate climate environments (St Clair et al. 2005). The primary objective of this study was to identify different adaptive strategies of tree populations, inferred from trait combinations and their associations with environmental variables. We use controlled environment common garden experiments to assess geographic variation in growth and adaptive traits. On this basis, we delineate genetically homogeneous groups of populations based on geographic and climatic criteria so that they can be used to manage adaptive variation under current and expected future climates. We further quantify among‐ and within‐population diversity using several variance partitioning approaches to assess how closely adaptive strategies of populations correspond to environmental gradients.

## Material and methods

### Seed source selection and characterization

**Figure 1:**
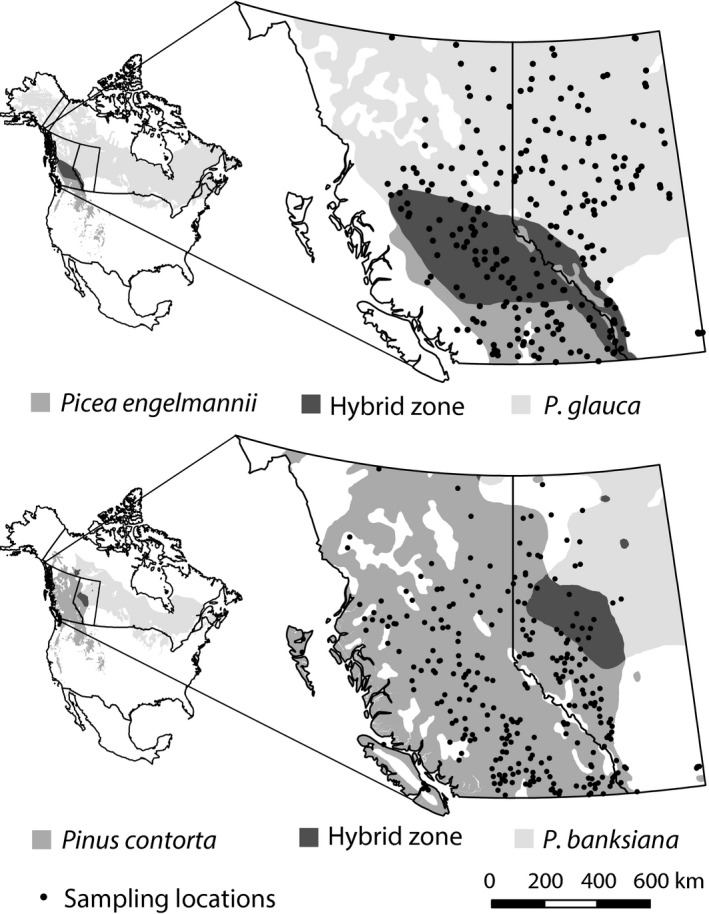
Origins of seedlots for common garden experiments with interior spruce and lodgepole pine. Species distributions and their overlap, representing areas of hybridization, are shown (Critchfield and Little 1966; Little 1971). *Picea glauca* and *Pinus banksiana* have boreal range distributions spanning the continent, while the ranges of *Picea engelmannii* and *Pinus contorta* are limited to western North America.

We obtained 254 bulk seedlots of interior spruce seed and 281 seedlots of lodgepole pine from natural populations (Fig. 1). Each seedlot contains open‐pollinated seeds from at least 10 female trees in British Columbia and at least 30 female trees in Alberta according to the provincial seed collection guidelines. We use the common name ‘interior spruce’ for a collectively managed species complex that includes white spruce (*P. glauca* [Moench] Voss), Engelmann spruce (*P. engelmannii* Parry ex Engelm.) and their natural hybrids (De La Torre et al. 2014). Lodgepole pine collections focus on the commercially important Rocky Mountain lodgepole pine subspecies (*P. contorta* Dougl. ex Loud. ssp. *latifolia* [Engelm.] Critchfield). However, we also include some samples from the coastal subspecies (*P. contorta* Dougl. ex Loud. ssp. *contorta*) and populations where the species hybridizes with jack pine (*Pinus banksiana* Lamb.) in Alberta.

Seedlots were selected based on ecosystem delineations as a proxy for different environmental conditions. We used ecosystem ‘variants’ of the hierarchical Biogeoclimatic Ecosystem Classification system for British Columbia (Meidinger and Pojar 1991), and the ecosystems of the Natural Regions and Subregions system at their finest level 4 for Alberta as ecosystem units (Natural Regions Committee 2006). Each ecosystem in which the species occurred was represented by two to six seedlots depending on its area and species frequency. For analytical purposes, we also use higher‐level summaries at the level of ‘ecological zones’ in British Columbia, approximately equivalent to ‘natural subregions’ in Alberta.

Seedlot origins were climatically characterized using the software package ClimateWNA (Hamann et al. 2013), available at http://tinyurl.com/ClimateWNA. We extracted climate normal data for the 1961–1990 period, which was chosen as a representation of climate prior to a significant anthropogenic warming signal, and because weather station data were most comprehensive for this period. The following biologically relevant climate variables were used in the analysis: mean annual temperature (MAT), mean warmest month temperature (MWMT), mean coldest month temperature (MCMT), continentality (TD, the temperature difference between MWMT and MCMT), degree‐days above 5°C (DD > 5), degree‐days below 0°C (DD < 0), mean annual precipitation, and May‐to‐September precipitation.

### Common garden experiments

Four common garden experiments with different temperature and moisture regimes were carried out per species in eight controlled environment chambers (Conviron, Winnipeg, MB, Canada). The growing conditions were programmed to emulate temperature regimes with MAT of 1°C, 6°C, and 11°C along a climate gradient from 55° to 46° latitude of the interior plateau of western North America. The 11°C temperature regime was applied to two experiments in different growth chambers with different moisture regimes, including a well‐watered treatment (as in the 1°C and 6°C chambers), and a drought treatment. Photoperiod simulated seasonal patterns for 54.5°N in all treatments. Seedlings were grown for two growing seasons equivalent to April 15 to October 15 climate conditions, and a compressed dormancy period of 6 weeks induced by an appropriate daylength regime and a constant temperature of 4°C. While growth chamber experiments can never truly emulate natural environments neither under current nor under projected future climate conditions, seedlings were exposed to a range of benign as well as stressful growing conditions. The objective was to detect population differentiation that may not be expressed under all environmental conditions. For details of the growth chamber programming, see Appendix S1 and Table S1.

The experimental design in each common garden experiment was an incomplete block *α*‐design implemented with the Alpha+ software package (Williams and Talbot 1993), which constructs a balanced design for any combination of treatment and block numbers. Each growth chamber had 720 seedlings per species that were distributed over eight incomplete blocks with 90 seedlings per block, for a total of 2880 seedlings in each of the interior spruce and lodgepole pine experiments. Therefore, each seedlot was represented by 2–4 seedlings per chamber (mean of 2.5 for lodgepole pine, 2.8 for interior spruce). Since we do not make inferences from how seedlings respond to these artificial environments, growth chambers are not replicated treatments and are considered random effects.

### Measurements

Height and root collar diameter were measured after the second growing season, when seedlings were dormant. At the beginning of the second season, budbreak was recorded in intervals of 2–3 days, starting in week two in the warmest chambers and week five in the coldest chambers. The monitoring of budset started in week 14 (~16 h daylength) and was recorded at intervals ranging from three to 10 days, depending on the observed rate of budset. The binary phenology data were transformed into a day of season (starting with an April 15 equivalent climate), where budbreak or budset was first observed.

Cold hardiness was assessed toward the end of the second growing season by artificial freeze testing using the electrolytic conductivity protocol described by Hannerz et al. (1999), carried out for three freezing temperatures. Test temperatures were chosen to result in intermediate levels of injury for each species–environment combination based on preliminary tests 1 week earlier. The selected freezing temperatures were −10°, −13°, and −16°C for lodgepole pine across all four experiments. Different temperatures were selected for interior spruce: −10°, −13°, and −16°C for the 1°C chamber; −16°, −20°, and −24°C for the 6°C chamber; and −10°, −14°, and −18°C for the two warm chambers. An index of injury was calculated for each sample as follows:

(1) $$I_{t}=\frac{100(R_{t}-R_{0})}{\left(1-R_{0}\right)}\;\text{with}\;R_{t}=\frac{L_{t}}{L_{k}}\;\text{and}\;R_{0}=\frac{L_{0}}{L_{d}}$$

where *I*_*t*_ is the index of injury (%), *R*_*t*_ is the relative conductance of the sample exposed to freeze temperature *t*, and *R*_0_ is the relative conductance of the control treatment. *L*_*t*_ is the conductance of leachate from the sample after freezing and *L*_*k*_ is the conductance of the leachate after the heat kill of the sample. *L*_0_ is the conductance of leachate from the unfrozen control and *L*_*d*_ is the conductance of the leachate after the heat kill of the unfrozen control.

Prior to analysis, cold hardiness data were summarized into a single frost hardiness variable by estimating least squares means of the two freeze treatments that resulted in close to 50% average damage across all populations and therefore provided maximum differentiation among populations (the additional third treatment did not improve correlations with climate of the origin of samples).

### Statistical analysis

Variance components and their standard errors were obtained using PROC MIXED with the option COVTEST of the SAS statistical software package (SAS Institute Inc. 2008) according to the following model:

(2) $$\begin{array}{c} Y_{ijkl}=\mu +P_{i}+E_{j}+{\left(P\times E\right)}_{ij}+B{\left(E\right)}_{jk}+L{\left(B\right)}_{kl}+e_{ijkl} \end{array}$$

where *Y*_*ijkl*_ is the phenotypic observation of a trait made for the *i*th population (*P*), with each seedlot sample representing a population, grown in the *j*th environment (*E*), located in the *k*th block (*B*) within environment *E* and at the *l*th location (*L*) within block. *P* × *E* represents the population by environment interaction, *μ* is the overall experimental mean, and *e* is the experimental error (residual). All model terms were considered random effects. The differentiation among populations (*V*_pop_) was calculated as $\sigma_{P}^{2}/\left(\sigma_{P}^{2}+\sigma_{e}^{2}\right)$, serving as a proxy for *Q*_ST_, where $\sigma_{P}^{2}$ is the variance among populations and $\sigma_{e}^{2}$ is the residual variance approximating the variance within populations (Alberto et al. 2013). *V*_pop_ is an underestimate of *Q*_ST_ because in the denominator the total phenotypic variance (among‐population component and residual error) is used, rather than the among‐population component and twice the additive variance component. Standard errors of *V*_pop_ were calculated according to standard rules for error propagation for addition (3) for the denominator ($\sigma_{P}^{2}+\sigma_{e}^{2}$) and division (4) for the ratio of the population over the total variance.

(3) $${\text{SE}}_{P+E}=\sqrt{{\text{SE}}_{P}^{2}+{\text{SE}}_{E}^{2}}$$

(4) $${\text{SE}}_{V_{\mathrm{pop}}}=V_{\mathrm{pop}}\times \sqrt{{\left(\frac{{\text{SE}}_{P}}{\sigma_{P}^{2}}\right)}^{2}+{\left(\frac{{\text{SE}}_{P+E}}{\sigma_{P}^{2}+\sigma_{e}^{2}}\right)}^{2}}$$

As an alternative variance partitioning approach, we also use multivariate regression tree (MRT) analysis, which is a constrained clustering method that partitions variance in one dataset (trait measurements for provenance) based on criteria of another dataset (climatic or geographic variables of provenance origins) according to De'Ath (2002). This analysis was conducted with the *MVpart* package version 1.6‐1 (R Development Core Team 2013).

Prior to this analysis, means of provenances were normalized, that is, expressed in units of standard deviations from the overall growth chamber mean, and missing values were imputed according to Hamann et al. (2011). As predictor variables, we use the climatic characterization of the seedlot origins, as well as ecosystem variants. The climatic variables were used to infer adaptation of local populations to their environments, while the same analysis with ecosystem variants as predictors was meant for assembling geographic zones that contain similar genotypes. For conciseness, we do not show results of individual chambers in the regression tree analysis results because the analysis of variance revealed weak interactions between population and environment. Instead, means across all chambers are shown in figures.

## Results

### Population and environmental variation

The analysis of variance indicated that main effects due to different growth chamber environments were large in most cases, with values up to 94% in the trait budbreak (Tables 1 and 2). Budbreak is directly controlled by heat sum accumulation and therefore simply reflects different temperature programming of chambers. Excluding the main effects of the growth chambers, the largest variance component in all traits of both species was the population main effect. The interaction term between population and environment (*P* × *E*) was substantially smaller than the population effect for interior spruce (about 12–40% of the population main effect) and largely absent in lodgepole pine (Table 1). The experimental design factors block and border within chambers generally accounted for little variance, reflecting relatively homogeneous growth chamber environments and the regular spatial rotation of blocks within chambers during the experiment.

**Table 1:** Variance components and the level of population differentiation (V_pop_) for five phenotypic traits of interior spruce

Source of variance	Variance components (%)				
	Height	Diameter	Budbreak	Budset	Cold injury
Population (*P*)	9.3 (1.3)	11.7 (1.8)	0.5 (0.1)	14.8 (2.1)	36.6 (4.1)
Environment (*E*)	35.9 (29.7)	13.1 (11.2)	93.3 (76.2)	17.2 (14.2)	0.3 (0.9)
*P *× *E*	3.9 (1.2)	3.9 (1.8)	0.2 (0.1)	5.4 (1.9)	4.5 (2.1)
Block (*B*) within *E*	3.1 (1.1)	4.0 (1.4)	0.2 (0.1)	0.6 (0.5)	2.4 (1.3)
Location (*L*) within *B*	1.4 (0.7)	2.3 (1.1)	0.1 (0.1)	0.0 (0.2)	3.9 (2.0)
Residual	46.4 (1.6)	65.1 (2.4)	5.6 (0.2)	62.0 (2.4)	52.3 (2.3)
*V*_pop_	16.6 (2.4)	15.2 (2.4)	7.4 (1.7)	19.3 (2.8)	41.1 (4.8)

The components are calculated based on a mixed model, with all factors being considered random effects. *V*_pop_ is given as proportion of the total phenotypic variation between populations. Standard errors are given in brackets.

**Table 2:** Variance components and the level of population differentiation (V_pop_) for five phenotypic traits of lodgepole pine

Source of variance	Variance components (%)				
	Height	Diameter	Budbreak	Budset	Cold injury
Population (*P*)	11.7 (1.6)	4.2 (1.0)	0.6 (0.1)	21.3 (2.7)	12.0 (1.9)
Environment (*E*)	28.1 (23.2)	19.3 (15.9)	93.9 (76.7)	1.9 (1.6)	12.5 (11.3)
*P *× *E*	2.0 (1.4)	0.0 (0.0)	0.3 (0.1)	0.0 (2.1)	0.0 (0.0)
Block (*B*) within *E*	1.5 (0.6)	0.8 (0.5)	0.1 (0.0)	0.2 (0.4)	5.5 (2.3)
Location (*L*) within *B*	6.5 (2.6)	2.3 (1.1)	0.0 (0.0)	0.2 (0.3)	2.5 (1.5)
Residual	50.1 (1.8)	73.4 (2.1)	5.1 (0.2)	76.4 (2.9)	67.6 (2.3)
*V*_pop_	19.0 (2.6)	5.4 (1.3)	10.9 (2.1)	21.8 (2.8)	15.0 (2.4)

The components are calculated based on a mixed model, with all factors being considered random effects. *V*_pop_ is given as proportion of the total phenotypic variation between populations. Standard errors are given in brackets.

**Figure 2:**
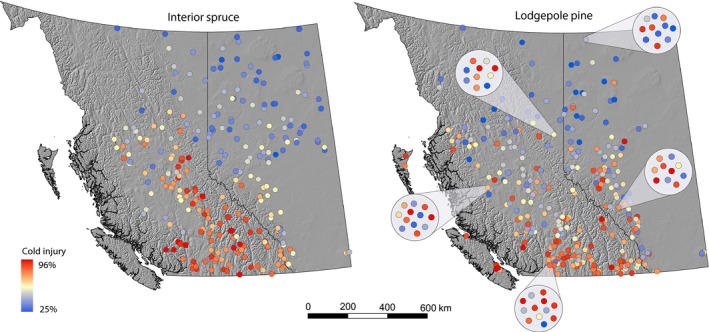
Cold injury of interior spruce and lodgepole pine populations. Each point on the landscape represents the average expression of all individuals from a given seedlot across all growth chamber environments. Five inserts for sample populations of lodgepole pine show the variation among individuals.

The among‐population variance (*V*_pop_) representing population differentiation as a proxy for *Q*_ST_ ranged from 7.7% to 41.1% in interior spruce and from 5.4% to 21.8% in lodgepole pine (Tables 1 and 2). The highest population differentiation was observed for cold injury in interior spruce (41.1%) and for timing of budset in lodgepole pine (21.8%). As a visual example for a strongly differentiated trait, Figure 2 shows geographically pronounced patterns for cold injury in interior spruce. As a visual example for a moderately differentiated trait, the same figure shows within‐ and among‐population differentiation of cold injury in pine. Although geographic population differentiation is clearly visible, it is also notable that some highly susceptible seedlings can be found in the far north, subarctic ecosystems of Alberta, while some very frost hardy individuals can be found in bulk seed collections from warm, southern British Columbia.

### Variance partitioning by climate of origin

**Figure 3:**
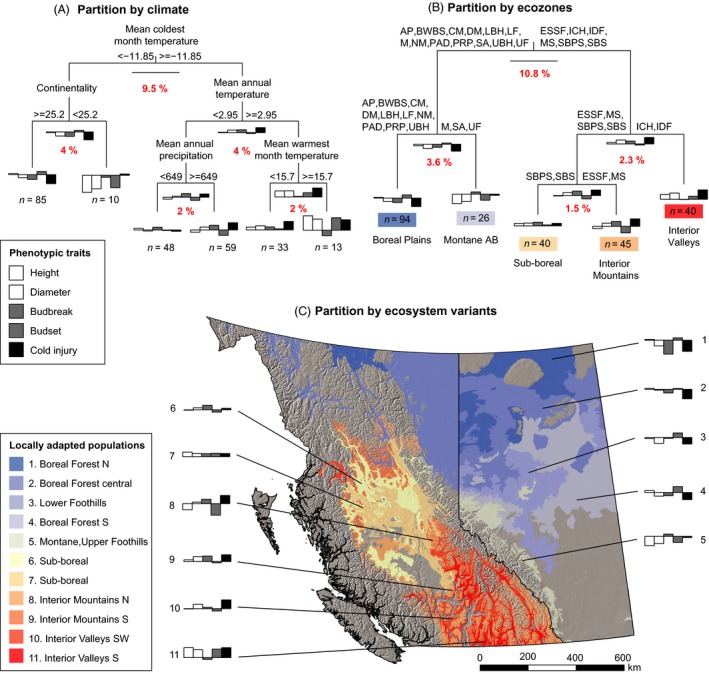
Geographic patterns of genetic adaptation to climate in interior spruce. Multivariate regression tree analysis was used to partition the genetic dataset by climate (A) and by ecozone (B). The spatial extent of the groups, resulting from the partition by ecosystem variant (C), is displayed across Alberta and British Columbia. Red numbers indicate the variance explained by a particular split. Bars in histograms at the end of the tree branches represent group means of phenotypic variation for each trait expressed in deviation from an overall mean of zero (horizontal line). Abbreviations in the ecozone partitioning (B) represent the second level of Alberta's Natural Regions and Subregions classification (A, Alpine; SA, Subalpine; M, Montane; CM, Central Mixedwood; DM, Dry Mixedwood; NM, Northern Mixedwood; BSA, Boreal Subarctic; PAD, Peace–Athabasca Delta; LBH, Lower Boreal Highlands; UBH, Upper Boreal Highlands; AP, Athabasca Plain; LF, Lower Foothills; UF, Upper Foothills; FP, Foothills Parkland; PRP, Peace River Parkland) and the ecological zones of British Columbia's Biogeoclimatic Ecosystem Classification (BWBS, Boreal White and Black Spruce; SBPS, Sub‐Boreal Pine–Spruce; SBS, Sub‐Boreal Spruce; ESSF, Engelmann Spruce–Subalpine Fir; MS, Montane Spruce; IDF, Interior Douglas‐fir; CDF, Coastal Douglas‐fir; ICH, Interior Cedar‐Hemlock; CWH, Coastal Western Hemlock).

Among‐population variation was further partitioned with a nonlinear approach, a MRT analysis. Using climate variables as partitioning criteria, populations of interior spruce split into six groups (Fig. 3A). Overall the tree explains 22% of the variance in the dataset, compared to an average of 20% in Table 2. The first split separates sources using the partitioning criterion MCMT, approximately describing a geographic separation by the Rocky Mountains. The leaf charts at the nodes of the dendrogram describe means for groups of similarly adapted seedlots in the five traits height, diameter, budbreak, budset, and cold injury. All trait values are expressed as standard deviation from an overall mean of zero, and therefore, upward bars indicate above‐average height, diameter, and cold injury, as well as later‐than‐average budbreak and budset.

To give an example for reading the information contained in Fig. 3A, spruce: the first node to the left represents 95 provenances from low minimum temperature origins, which show average height growth, slightly below‐average diameter, early budbreak, approximately average budset, and low cold injury. The subsequent split to the right singles out a subset of 10 provenances from this group with slow growth and early budset that are characterized by low winter minimum temperatures but in addition also by a less continental climate (driven by cool summer temperatures—these are high‐elevation provenances from Alberta). The remaining 85 sources come from the boreal plains. Following all splits toward the right, we arrive at a provenance group from environments with warm winter, annual, and summer temperatures. Those provenances originate from the interior valleys of southern British Columbia, and they are characterized by very high growth, long growing season utilization, but also high cold injury. The remaining groups are 33 provenances from the interior plateau of British Columbia with cooler summers, 59 provenances from higher elevations with higher precipitation, and a group of 48 provenances from interior British Columbia from overall cooler and drier locations.

Using ecozones as partitioning criteria (Fig. 3B), we obtain similar results as in Fig. 3A with groups that represent five major geographic regions: the boreal plains, montane Alberta, the sub‐boreal, the interior mountains, and the interior valleys of British Columbia. Further subdivision of the groups, using 115 ecosystem variants as partitioning criteria, results in 11 groups for spruce. Notable subgroups are provenances from the boreal subarctic with little cold injury and early budbreak in common gardens (Fig. 3C, group 1), and montane populations, characterized by a short growing period with late budbreak and early budset (groups 5, 8, and 9).

**Figure 4:**
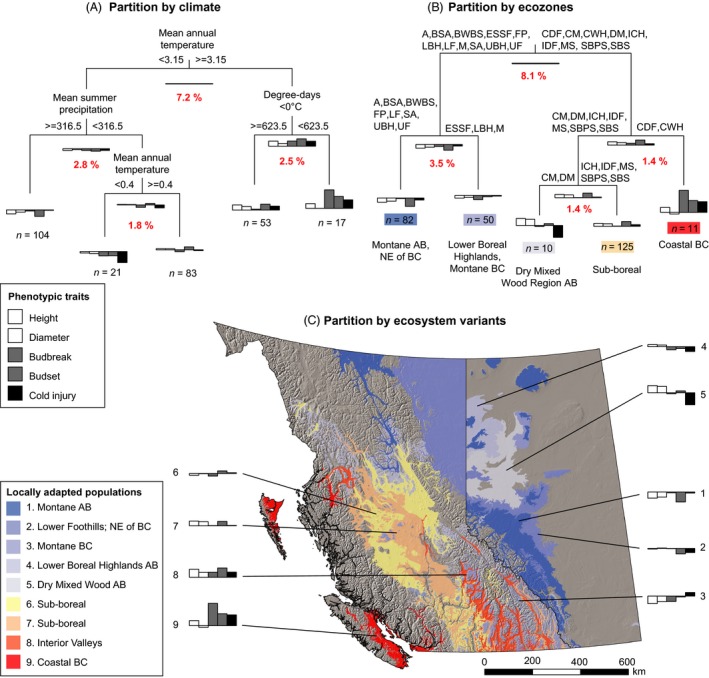
Geographic patterns of genetic adaptation to climate in lodgepole pine. Multivariate regression tree analysis was used to partition the genetic dataset by climate (A) and by ecozone (B). The spatial extent of the groups, resulting from the partition by ecosystem variant (C), is displayed across Alberta and British Columbia. Red numbers indicate the variance explained by a particular split. Bars in histograms at the end of the tree branches represent group means of phenotypic variation for each trait expressed in deviation from an overall mean of zero (horizontal line). Explanations for the abbreviations in the partitioning by ecozone are given in Fig. 3.

Lodgepole pine reveals adaptive trait combinations similar to those found in spruce, and genetic differentiation is mainly driven by temperature variables, except for high‐elevation sources that are partitioned out by high precipitation (far left group in Fig. 4A,B). Sources of the subspecies *contorta* from coastal BC with late budbreak, late budset, and high cold injury (outside the range of interior spruce) form an additional uniquely adapted group (far right groups in Fig. 4A,B).

## Discussion

### General types of adaptive strategies to climate

Across both species, we find multitrait combinations that were consistent, regardless of whether climate or geographic delineations were used as partitioning criteria. Populations adapted to a continental climate with harsh winters across the boreal plains combine high cold hardiness with early budbreak and late budset. Early budbreak is a suitable adaptation for environments with rapid transitions from cold temperatures to favorable growing conditions in spring. Under the most northern boreal conditions for spruce (group 1 in Fig. 3C), where the growing season is most limited, this trait combination is most pronounced, with an extremely early budbreak for a given heat sum in a controlled environment. In pine, the populations closest to boreal conditions (groups 4 and 5, Fig. 4C) show similar tendencies in cold hardiness and phenology to spruce. These populations take a higher risk of late spring frost damage to fully utilize the short available growing season in the far north.

Montane populations of spruce (groups 5, 8, and 9 in Fig. 3C and pine groups 1 and 3 in Fig. 4C) show a different combination of growth and adaptive traits in a common garden environment. All high‐elevation sources show strongly contrasting phenology and hardiness to boreal populations. They break bud late, set bud early, do not have strong tolerance to cold, and tend to be poor growers. We interpret this trait combination as an adaptation to a small number of growing degree‐days and a long‐lasting, deep snowpack that provides protection against very low temperatures (De La Torre et al. 2014). Their early budset timing at daylengths where lower‐elevation populations keep growing reflects an adaptation to short periods suitable for growth. In our common garden setting, the limited utilization of the available growing season puts high‐elevation sources at a competitive disadvantage in both species.

Populations of both species from the sub‐boreal interior plateau of British Columbia represent average genotypes in our study. Genotypes start to divert from this average toward the southern interior valleys in spruce and pine, and toward the coastal range of pine where the species experiences the warmest and most maritime climate conditions. Lower‐elevation populations from interior valleys typically grew best. These provenances experience extended growing seasons at their location of origin, and they also grew longer in the common garden setting than other provenances in both species. This growth pattern likely reflects an allocation of resources to compete for light in more favorable growing environments with higher interspecies competition. Investments in cold hardiness are low in these regions.

Shore pine populations from coastal environments (*P. contorta* ssp. *contorta*) stand out from interior Rocky Mountain lodgepole pine (ssp. *latifolia*) by having an exceptionally late budbreak, potentially caused by an additional chilling requirement that needs to be fulfilled before heat sum accumulation in spring. High chilling requirements are common for species or provenances from maritime climates as, for example, observed in Sitka spruce (*Picea sitchensis*) (Cannell and Smith 1983; Hannerz et al. 2003) or Douglas‐fir (*Pseudotsuga menziesii*) (Campbell and Sugano 1979). They prevent trees from flushing mid‐winter due to regular occurrences of temperatures adequate for forcing.

A notable result of this analysis is that adaptation to climate does not usually correspond linearly to climate gradients in this study. For example, opposite trait values (e.g., early versus late budbreak) may be found in response to apparently similar cold environments (e.g., boreal and montane). As a result, simple correlations with climate variables are generally quite low, with correlation coefficients between zero and 0.4 except for frost hardiness (Tables S2 and S3). Similarly, linear correlations among measured traits also tend to be very low (Tables S4 and S5). We propose that such low correlations do not necessarily imply lack of local adaptation or lack of tradeoffs among growth and adaptive traits. Rather, when sampling spans complex environments rather than simple gradients, analytical tools that account for complex interactions among multiple traits and environments are required, also observed by St Clair et al. (2005). In our case, MRT analysis was able to identify consistent adaptive syndromes with similar trait combinations in spruce and pine (e.g., for montane vs. boreal vs. warm and dry environments), while simple trait correlations are not necessarily identical because of different species ranges and resultant sampling of environments [e.g., budset is positively correlated with MCMT in pine, but not in spruce (Tables S2 and S3)].

### Within‐ and among‐population diversity

Although not unusual in forest trees, this study documented relatively high levels of within‐population diversity in seedling growth and adaptive traits, and somewhat low among population differentiation. Our results are toward the low end of population differentiation observed in a recent review of Alberto et al. (2013). Population differentiation measured as *Q*_ST_ or *V*_pop_ in 13 North American conifers was on average 0.34 for height, 0.25 for budbreak, 0.34 for budset, and 0.68 for fall cold hardiness. Previous estimates for lodgepole pine were also higher with 0.57 for fall cold hardiness and 0.45 for height (Rehfeldt 1980; Chuine et al. 2001). *Q*_ST_ estimates for white spruce in eastern Canada, however, are closer to our estimates with 0.25 for budset and 0.08 for 8‐year height (Jaramillo‐Correa et al. 2001). *Q*_ST_ estimates are variable depending on the range and number of populations sampled, and our relatively low values may be explained by a relatively dense sampling of only part of the range of the species. It should be noted as well that *V*_pop_ is an underestimate of *Q*_ST_ as explained in the Material and methods section.

This high within‐population genetic diversity is likely to be maintained across the heterogeneous forest landscape by gene flow (Yeaman and Jarvis 2006; Kremer et al. 2012). Although we cannot assess adaptive capacity for lack of pedigree information in our provenance experiments with bulk seed lots, the high degree of within‐population diversity does not suggest a lack of evolutionary capacity to adapt to new environmental conditions through natural selection. The analysis also implies that assisted gene flow prescriptions to address climate change do not need to match populations to environments at very fine spatial or climatic scales.

### Management applications in reforestation

To manage seed transfer in forestry operations, 21 seed zones for natural seedlots are in use for interior British Columbia (Ying and Yanchuk 2006). Within each zone, seed can be transferred from collection to any planting locations within additional species‐specific elevational, latitudinal, and longitudinal limits (Snetsinger 2005). In Alberta, a total of 44 seed zones are in use for natural seedlots of white spruce and 34 for lodgepole pine (ESRD 2009). Deployment zones for selectively bred planting stock produced through local breeding programs tend to be larger, because the environmental tolerances of these genotypes have been well studied in field‐based multisite progeny trials. British Columbia uses 16 zones for selectively bred lodgepole pine and 11 zones for interior spruce (Snetsinger 2005), while Alberta uses six and nine zones, respectively.

Our data suggest that such large numbers of seed zones for collections from natural stands may not be necessary. Moderately strong among‐population variation in important adaptive traits (Figs 3 and 4) and equivalent growth data from long‐term growth data from field trials (Wang et al. 2006; Rweyongeza et al. 2007, 2010) suggest that geographic restrictions to seed transfer on a relatively broad scale are sufficient. We propose that seed zone delineations for genetically improved planting stock would be appropriate to use with seed from natural stands as well. The assemblages of ecosystem variants according to MRT analysis (see Tables S6 and S7) quite closely align with existing breeding zone delineations, and breeding zone delineations used as a predictor variable in MRT analysis explain almost exactly the same amount of variance: 20% in interior spruce and 13% in lodgepole pine (analysis not shown).

Geographic restrictions to seed transfer via seed zones or seed transfer guidelines are standard reforestation practice in western North America to avoid maladaptation of reforestation stock (e.g., Hamann et al. 2011). Such delineations, however, make the foundational assumptions of optimal local adaptation, and our delineations of locally adapted populations are no exception. Local optimality may not apply for various reasons including the migration history of tree populations and recent environmental change (e.g., Mátyás 1994). Projected climate change may further increase the mismatch between populations and the environment to which they are optimally adapted. One way to address the problem is to use climate niche modeling techniques to project where matching multivariate climate habitat are expected in the future (e.g., Gray et al. 2011). Assisted migration prescriptions based on such projections would perpetuate any existing adaptational lag, but they can counter increasing maladaptation due to climate change.

## Author contributions

S.N.A. and A.H. conceived and designed the study, and contributed analysis tools and data. P.S. developed all treatment regimes and organized data collection. C.R.F. designed and established the growth chamber experiments. K.J.L. performed the analysis and wrote the manuscript. A.H., S.N.A., P.S., and C.R.F. revised the manuscript.

## Data Archiving Statement

Data for this study are available at the genome database Dendrome. Download links for lodgepole pine data are available at: https://dendrome.ucdavis.edu/ftgsc/population_set_info.php?inv_subprojects_id=93 Download links for interior spruce data are available at: https://dendrome.ucdavis.edu/ftgsc/population_set_info.php?inv_subprojects_id=92

## Supporting information


**Appendix S1.** Detailed method description for growth chamber experiments.
**Table S1.** Photo‐ and thermoperiodic growth chamber regimes of the intermediate chamber (6°C) during the second growing season.
**Table S2.** Pearson correlation coefficients* between traits measured in four growth chambers and the climate and geographic variables of the source locations of interior spruce provenances.
**Table S3.** Pearson correlation coefficients* between traits measured in four growth chambers and the climate and geographic variables of the source locations of lodgepole pine provenances.
**Table S4.** Pearson correlation coefficients* among traits measured in four growth chambers for interior spruce provenances.
**Table S5.** Pearson correlation coefficients* among traits measured in four growth chambers for lodgepole pine provenances.
**Table S6.** Groups of similarly adapted seedlots of interior spruce, derived by multivariate regression tree analysis based on ecosystem variants, corresponding to the groups in Fig. 3C.
**Table S7.** Groups of similarly adapted seedlots of lodgepole pine, derived by the multivariate regression tree analysis based on ecosystem variants, corresponding to the groups in Fig. 4C.
