# Supplementary material for: Adaptation of lodgepole pine and interior spruce to climate: implications for reforestation in a warming world
Source: Evol Appl. 2016 Jan 19;9(2):409–19. doi: 10.1111/eva.12345 (PMC4721073; doi:10.1111/eva.12345)
Supplement: Supplementary file 1 — Appendix S1. Detailed method description for growth chamber experiments. Table S1. Photo‐ and thermoperiodic growth chamber regimes of the intermediate chamber (6°C) during the second growing season. Table S2. Pearson correlation coefficients* between traits measured in four growth chambers and the climate and geographic variables of the source locations of interior spruce provenances. Table S3. Pearson correlation coefficients* between traits measured in four growth chambers and the climate and geographic variables of the source locations of lodgepole pine provenances. Table S4. Pearson correlation coefficients* among traits measured in four growth chambers for interior spruce provenances. Table S5. Pearson correlation coefficients* among traits measured in four growth chambers for lodgepole pine provenances. Table S6. Groups of similarly adapted seedlots of interior spruce, derived by multivariate regression tree analysis based on ecosystem variants, corresponding to the groups in Fig. 3C. Table S7. Groups of similarly adapted seedlots of lodgepole pine, derived by the multivariate regression tree analysis based on ecosystem variants, corresponding to the groups in Fig. 4C. [file EVA-9-409-s001.docx]

**Supporting Information for Liepe et al. Adaptation of lodgepole pine and interior spruce to climate: implications for reforestation in a warming world.**

**Appendix S1** Detailed method description for growth chamber experiments

*Nursery procedures*

Prior to sowing, seeds were soaked in distilled H_2_O for 24 h, disinfected with a one-minute rinse in 1.5% sodium hypochlorite bleach solution, surface dried and then stratified for approximately 5 weeks at 4°C. Seeds were sown in 49 ml single-tree containers (Stuewe & Sons. Inc., Oregon, USA) in a mixture of peat moss (75%) and perlite (25%), and covered with 5–8 mm of ‘forestry sand’ (Target Products Ltd., Alberta, Canada) to prevent drying out. Based on germination tests, seed were double or triple sown, and thinned if needed. To induce germination, seed were kept for two weeks in a greenhouse with temperatures of 24/20°C (day/night), with moisture being applied by misting (three times a week). The seedlings were then transferred to the four growth chambers regimes, where they were grown for two seasons. During the dormancy period, plant plugs were transplanted into trays (36 × 40 cm, giving each seedling a space of 4 × 4 cm) to allow for more growing space. The soil mixture used in the trays contained peat moss (50%), fine bark (25%) and fine pumice (25%) to facilitate drought cycles.

*Temperature programming*

The temperature regimes for the growth chambers were designed to approximate growing conditions along a north-south climate gradient of the interior plateau of western North America, ranging from Mackenzie, (1°C MAT, 55°N) to Merritt, (6°C MAT, 50°N) to Lewiston, (11°C MAT, 46°N). Monthly averages of the climate normal period (1961-1990) for target locations were extracted from ClimateWNA ([*http://tinyurl.com/ClimateWNA*](http://tinyurl.com/ClimateWNA)), averaged, and interpolated to weekly averages for a period from mid-April to mid-October. This 25 week growing season was used as a baseline climate for the intermediate chamber (6°C). A daily sinusoidal pattern was overlaid on the seasonal variation in two alternating phases: a 3-day warm phase with large temperature variation, mimicking sunny days and clear nights, and a 4-day cool phase with smaller diurnal fluctuations to mimic cloudy days and nights. The average weekly diurnal variation increased from 12°C at the beginning of the growing season to 15°C in the summer, and back to 12°C in the fall. However, daily diurnal variation varied between 8° and 23°C with the smallest values during the cold phases in spring and fall, and the highest values during simulated sunny phases in the summer. An example of the temperature regime for the intermediate chamber (6°C) is provided in Table S1. The climates for the cold (1°C) and warm chambers (11°C) were obtained by adding and subtracting 5°C from the established baseline climate of the intermediate chamber (6°C).

*Photoperiod and winter season*

A photoperiod regime equivalent to 54.5° latitude was applied across all chambers. This represents the central latitude of the two provinces and important commercial forestry areas for lodgepole pine and interior spruce. Photoperiod increased in weekly intervals from 14 h of light in week one to 17 h in week 10 and dropped back to 10:30 h in week 25 (Table S1). The first period of winter dormancy between first and second season was substituted by a compacted six week chilling period (compared to 27 weeks under natural conditions) with 8 h day length and a constant temperature of 4°C. To promote the development of cold hardiness at the end of the second growing season, a five-day period of frost treatments was applied around week 21. Nighttime minima were gradually ramped down to –2°C (the coldest setting of the growth chambers) for 2 h on the fifth day of the freezing treatments. While we could induce bud dormancy and cold hardiness development with this temperature and day length regime, it was not possible to simulate realistic frost regimes, which would also rely on appropriate snow cover and soil temperatures.

*Water and nutrient regime*

All treatments were well watered in biweekly intervals throughout the first season. During the second growing season seedlings in the warm and dry treatment (11°C dry) were subjected to drought cycles (14 cycles for spruce and 20 for pine), while all other treatments remained well watered. Consistent water regimes were applied according to tray weights. In spring and fall all pine treatments were watered every five days, while spruce treatments were watered every six days, by adding water until the trays reached a weight of approximately 14 kg for well-watered treatments, and 10 kg for the dry treatments. In the simulated summer month well-watered treatments were watered a second time in the middle of each cycle. Following these guidelines, the soil water content of well-watered treatments never dropped below 65% relative to saturation, while the dry treatments experienced a minimum soil water content of 25% relative to saturation. This level had previously been established as corresponding to a soil water potential of –1MPa, a level below which permanent damage starts to appear. In the first season fertilizer was applied with every watering (20-8-20 N-P-K, at 1g/L). During the second season fertilizer was still applied equally across all treatments, i.e. during the summer well-watered treatments received fertilizer with every second watering, while the drought treatments received fertilizer with every watering (20-8-20 N-P-K, at 2g/L to compensate for a decreased frequency).

**Table S1** Photo- and thermoperiodic growth chamber regimes of the intermediate chamber (6°C) during the second growing season. Weeks were seven days long, with a 3-day warm phase, followed by a 4-day cold phase. Each day, temperatures varied in a sinusoidal pattern, with maximum temperatures at 1 p.m. (max) and minimum temperatures at
1 a.m. (min)

|  |  | Temperature (°C) | | | | | |
| --- | --- | --- | --- | --- | --- | --- | --- |
| Week | Photoperiod (h) | Weekly average | Warm days | |  | Cold days | |
|  |  |  | max | min |  | max | min |
| 1 | 13:59 | 6.1 | 13.8 | 1.8 |  | 10.8 | 0 |
| 2 | 14:29 | 7.2 | 15.4 | 2.4 |  | 11.9 | 0 |
| 3 | 15:10 | 8.1 | 17.9 | 2.9 |  | 12.9 | 0 |
| 4 | 15:37 | 9.2 | 19.4 | 3.4 |  | 13.9 | 0.9 |
| 5 | 16:07 | 10.1 | 21.4 | 4.4 |  | 13.9 | 1.9 |
| 6 | 16:29 | 10.9 | 23.3 | 5.3 |  | 13.8 | 2.8 |
| 7 | 16:52 | 11.7 | 25.7 | 5.7 |  | 14.2 | 3.2 |
| 8 | 17:06 | 12.8 | 27.3 | 6.3 |  | 14.8 | 4.8 |
| 9 | 17:14 | 13.6 | 29.2 | 7.2 |  | 14.7 | 5.7 |
| 10 | 17:16 | 14.4 | 30.0 | 8.0 |  | 15.0 | 7.0 |
| 11 | 17:10 | 15.2 | 31.3 | 8.3 |  | 16.3 | 7.3 |
| 12 | 17:00 | 15.8 | 31.9 | 8.9 |  | 16.9 | 7.9 |
| 13 | 16:42 | 16.1 | 32.7 | 9.7 |  | 16.7 | 7.7 |
| 14 | 16:21 | 16.3 | 32.9 | 9.9 |  | 16.9 | 7.9 |
| 15 | 15:51 | 16.3 | 32.9 | 9.9 |  | 16.9 | 7.9 |
| 16 | 15:25 | 16.2 | 32.8 | 9.8 |  | 16.8 | 7.8 |
| 17 | 14:53 | 15.9 | 32.0 | 9.0 |  | 17.0 | 8.0 |
| 18 | 14:25 | 15.0 | 31.1 | 8.1 |  | 16.1 | 7.1 |
| 19 | 13:46 | 13.8 | 28.3 | 7.3 |  | 14.3 | 7.3 |
| 20 | 13:16 | 12.4 | 26.9 | 5.9 |  | 12.9 | 5.9 |
| 21 | 12:42 | 11.0 | 25.5 | 4.5 |  | 11.5 | 4.5 |
| 22 | 12:11 | 9.6 | 23.1 | 4.1 |  | 10.6 | 2.6 |
| 23 | 11:36 | 8.4 | 19.3 | 4.3 |  | 9.8 | 1.8 |
| 24 | 11:06 | 7.0 | 17.4 | 3.4 |  | 8.9 | 0 |
| 25 | 10:31 | 5.6 | 15.5 | 2.5 |  | 8.0 | 0 |

**Table S2** Pearson correlation coefficients* between traits measured in four growth chambers and the climate and geographic variables of the source locations of interior spruce provenances.

|  | Source climate | | | | | | | |  | Geography | |
| --- | --- | --- | --- | --- | --- | --- | --- | --- | --- | --- | --- |
|  | MAT | MWMT | MCMT | TD | DD>5 | DD<0 | log(MAP) | log(MSP) |  | Latitude | Elevation |
| Interior spruce 1°C |  |  |  |  |  |  |  |  |  |  |  |
| Diameter | 0.224 | -0.017 | 0.212 | -0.186 | 0.017 | -0.221 | 0.228 | -0.074 |  | -0.186 | 0.037 |
| Height | 0.262 | 0.355 | 0.036 | 0.062 | 0.407 | -0.086 | -0.069 | -0.222 |  | -0.003 | -0.335 |
| Budbreak | 0.073 | -0.435 | 0.273 | -0.347 | -0.420 | -0.252 | 0.298 | 0.172 |  | -0.227 | 0.385 |
| Budset | 0.100 | 0.538 | -0.215 | 0.325 | 0.565 | 0.149 | -0.400 | -0.298 |  | 0.181 | -0.503 |
| Cold injury | 0.540 | -0.233 | 0.591 | -0.566 | -0.142 | -0.607 | 0.510 | 0.177 |  | -0.569 | 0.377 |
|  |  |  |  |  |  |  |  |  |  |  |  |
| Interior spruce 6°C |  |  |  |  |  |  |  |  |  |  |  |
| Diameter | 0.314 | 0.037 | 0.263 | -0.216 | 0.077 | -0.288 | 0.163 | -0.157 |  | -0.215 | 0.012 |
| Height | 0.360 | 0.273 | 0.176 | -0.079 | 0.333 | -0.221 | 0.010 | -0.266 |  | -0.103 | -0.231 |
| Budbreak | 0.177 | -0.211 | 0.267 | -0.284 | -0.186 | -0.254 | 0.173 | 0.152 |  | -0.240 | 0.270 |
| Budset | 0.192 | 0.387 | -0.036 | 0.132 | 0.423 | -0.007 | -0.132 | -0.141 |  | 0.064 | -0.339 |
| Cold injury | 0.650 | -0.289 | 0.735 | -0.704 | -0.188 | -0.744 | 0.633 | 0.169 |  | -0.732 | 0.485 |
|  |  |  |  |  |  |  |  |  |  |  |  |
| Interior spruce 11°C |  |  |  |  |  |  |  |  |  |  |  |
| Diameter | 0.224 | 0.034 | 0.201 | -0.163 | 0.051 | -0.211 | 0.171 | -0.133 |  | -0.151 | 0.008 |
| Height | 0.122 | 0.197 | -0.001 | 0.052 | 0.215 | -0.030 | -0.039 | -0.221 |  | 0.051 | -0.218 |
| Budbreak | 0.397 | -0.253 | 0.465 | -0.464 | -0.185 | -0.475 | 0.328 | 0.155 |  | -0.398 | 0.312 |
| Budset | 0.214 | 0.381 | -0.031 | 0.127 | 0.424 | -0.026 | -0.166 | -0.180 |  | 0.070 | -0.367 |
| Cold injury | 0.355 | -0.240 | 0.471 | -0.466 | -0.200 | -0.457 | 0.493 | 0.188 |  | -0.470 | 0.388 |
|  |  |  |  |  |  |  |  |  |  |  |  |
| Interior spruce 11°C, dry |  |  |  |  |  |  |  |  |  |  |  |
| Diameter | 0.271 | -0.014 | 0.253 | -0.220 | 0.014 | -0.271 | 0.113 | -0.165 |  | -0.163 | 0.028 |
| Height | -0.133 | 0.267 | -0.278 | 0.308 | 0.250 | 0.244 | -0.341 | -0.229 |  | 0.334 | -0.404 |
| Budbreak | 0.182 | -0.240 | 0.242 | -0.270 | -0.186 | -0.254 | 0.118 | 0.089 |  | -0.167 | 0.203 |
| Budset | -0.058 | 0.243 | -0.221 | 0.250 | 0.268 | 0.182 | -0.356 | -0.196 |  | 0.319 | -0.394 |
| Cold injury | 0.375 | -0.201 | 0.461 | -0.443 | -0.154 | -0.450 | 0.487 | 0.152 |  | -0.450 | 0.338 |

* Correlation coefficients equal to or greater than 0.204 are significantly different from zero for df=252 and a corrected α=0.0011 after a sequential Bonferroni adjustment for multiple inferences (k=10).

**Table S3** Pearson correlation coefficients* between traits measured in four growth chambers and the climate and geographic variables of the source locations of lodgepole pine provenances.

|  | Source climate | | | | | | | |  | Geography | |
| --- | --- | --- | --- | --- | --- | --- | --- | --- | --- | --- | --- |
|  | MAT | MWMT | MCMT | TD | DD>5 | DD<0 | log(MAP) | log(MSP) |  | Latitude | Elevation |
| Chamber 1°C |  |  |  |  |  |  |  |  |  |  |  |
| Diameter | 0.161 | 0.211 | 0.070 | 0.008 | 0.212 | -0.098 | -0.101 | -0.125 |  | -0.055 | -0.135 |
| Height | 0.338 | 0.419 | 0.139 | 0.016 | 0.446 | -0.200 | -0.108 | -0.225 |  | -0.025 | -0.362 |
| Budbreak | 0.542 | 0.264 | 0.478 | -0.375 | 0.362 | -0.515 | 0.216 | -0.251 |  | -0.232 | -0.325 |
| Budset | 0.398 | 0.190 | 0.338 | -0.250 | 0.241 | -0.378 | 0.025 | -0.361 |  | -0.190 | -0.210 |
| Cold injury | 0.334 | 0.033 | 0.394 | -0.376 | 0.091 | -0.402 | 0.320 | -0.032 |  | -0.427 | 0.097 |
|  |  |  |  |  |  |  |  |  |  |  |  |
| Chamber 6°C |  |  |  |  |  |  |  |  |  |  |  |
| Diameter | 0.100 | 0.239 | -0.038 | 0.125 | 0.224 | -0.012 | -0.102 | -0.095 |  | 0.040 | -0.189 |
| Height | 0.246 | 0.350 | 0.069 | 0.060 | 0.361 | -0.125 | -0.082 | -0.181 |  | 0.039 | -0.345 |
| Budbreak | 0.230 | 0.139 | 0.180 | -0.127 | 0.208 | -0.172 | 0.187 | 0.137 |  | 0.039 | -0.241 |
| Budset | 0.300 | 0.243 | 0.203 | -0.104 | 0.259 | -0.254 | 0.030 | -0.331 |  | -0.107 | -0.246 |
| Cold injury | 0.353 | 0.085 | 0.409 | -0.372 | 0.129 | -0.407 | 0.254 | -0.031 |  | -0.421 | 0.084 |
|  |  |  |  |  |  |  |  |  |  |  |  |
| Chamber 11°C |  |  |  |  |  |  |  |  |  |  |  |
| Diameter | 0.277 | 0.251 | 0.180 | -0.085 | 0.279 | -0.216 | 0.069 | -0.144 |  | -0.065 | -0.217 |
| Height | 0.273 | 0.267 | 0.162 | -0.063 | 0.303 | -0.192 | 0.066 | -0.133 |  | 0.010 | -0.302 |
| Budbreak | 0.155 | 0.120 | 0.092 | -0.047 | 0.183 | -0.077 | 0.183 | 0.127 |  | 0.106 | -0.288 |
| Budset | 0.368 | 0.232 | 0.296 | -0.206 | 0.283 | -0.336 | 0.091 | -0.332 |  | -0.160 | -0.276 |
| Cold injury | 0.256 | 0.081 | 0.288 | -0.256 | 0.123 | -0.273 | 0.199 | 0.014 |  | -0.306 | 0.062 |
|  |  |  |  |  |  |  |  |  |  |  |  |
| Chamber 11°C, dry |  |  |  |  |  |  |  |  |  |  |  |
| Diameter | 0.091 | 0.134 | 0.012 | 0.037 | 0.143 | -0.048 | -0.109 | -0.145 |  | 0.014 | -0.142 |
| Height | 0.141 | 0.183 | 0.050 | 0.017 | 0.185 | -0.090 | -0.067 | -0.218 |  | 0.015 | -0.207 |
| Budbreak | 0.254 | 0.130 | 0.204 | -0.154 | 0.212 | -0.194 | 0.232 | 0.150 |  | 0.027 | -0.272 |
| Budset | 0.390 | 0.231 | 0.323 | -0.231 | 0.275 | -0.368 | 0.140 | -0.344 |  | -0.233 | -0.245 |
| Cold injury | 0.364 | 0.082 | 0.417 | -0.379 | 0.131 | -0.413 | 0.233 | -0.067 |  | -0.410 | 0.095 |

* Correlation coefficients equal to or greater than 0.194 are significantly different from zero for df=279 and a corrected α=0.0011 after a sequential Bonferroni adjustment for multiple inferences (k=10).

**Table S4.** Pearson correlation coefficients* among traits measured in four growth chambers for interior spruce provenances.

|  | Diameter | Budbreak | Budset | Cold injury |
| --- | --- | --- | --- | --- |
| Chamber 1°C |  |  |  |  |
| Height | 0.554 | -0.239 | 0.519 | 0.080 |
| Diameter |  | 0.020 | -0.095 | -0.002 |
| Budbreak |  |  | -0.351 | 0.145 |
| Budset |  |  |  | 0.052 |
|  |  |  |  |  |
| Chamber 6°C |  |  |  |  |
| Height | 0.717 | -0.005 | 0.389 | 0.101 |
| Diameter |  | 0.082 | 0.082 | 0.128 |
| Budbreak |  |  | -0.052 | 0.121 |
| Budset |  |  |  | -0.033 |
|  |  |  |  |  |
| Chamber 11°C |  |  |  |  |
| Height | 0.657 | 0.025 | 0.553 | -0.101 |
| Diameter |  | 0.099 | 0.362 | 0.040 |
| Budbreak |  |  | -0.038 | 0.089 |
| Budset |  |  |  | -0.075 |
|  |  |  |  |  |
| Chamber 11°C, dry |  |  |  |  |
| Height | 0.372 | -0.009 | 0.505 | -0.248 |
| Diameter |  | 0.146 | 0.198 | 0.094 |
| Budbreak |  |  | -0.026 | 0.044 |
| Budset |  |  |  | -0.100 |

* Correlation coefficients equal to or greater than 0.123 are significantly different from zero for df= 252 and α=0.05.

**Table S5.** Pearson correlation coefficients* among traits measured in four growth chambers for lodgepole pine provenances.

|  | Diameter | Budbreak | Budset | Cold injury |
| --- | --- | --- | --- | --- |
| Lodgepole pine 1°C |  |  |  |  |
| Height | 0.602 | 0.393 | 0.284 | -0.030 |
| Diameter |  | 0.172 | 0.149 | -0.123 |
| Budbreak |  |  | 0.515 | 0.250 |
| Budset |  |  |  | 0.199 |
|  |  |  |  |  |
| Lodgepole pine 6°C |  |  |  |  |
| Height | 0.707 | 0.287 | 0.206 | -0.336 |
| Diameter |  | 0.100 | 0.106 | -0.375 |
| Budbreak |  |  | -0.068 | -0.047 |
| Budset |  |  |  | 0.121 |
|  |  |  |  |  |
| Lodgepole pine 11°C |  |  |  |  |
| Height | 0.645 | 0.120 | 0.317 | -0.235 |
| Diameter |  | -0.027 | 0.308 | -0.201 |
| Budbreak |  |  | -0.115 | 0.051 |
| Budset |  |  |  | -0.023 |
|  |  |  |  |  |
| Lodgepole pine 11°C, dry | |  |  |  |
| Height | 0.559 | 0.059 | 0.291 | -0.246 |
| Diameter |  | 0.002 | 0.165 | -0.261 |
| Budbreak |  |  | -0.056 | -0.003 |
| Budset |  |  |  | 0.127 |

* Correlation coefficients equal to or greater than 0.117 are significantly different from zero for df= 279 and α=0.05.

Table S6. Groups of similarly adapted seedlots of interior spruce, derived by multivariate regression tree analysis based on ecosystem variants, corresponding to the groups in Fig. 3c

|  |  | Ecosystem variants by province‡ | | | | | | | |
| --- | --- | --- | --- | --- | --- | --- | --- | --- | --- |
| Group* | Group name* | Alberta | | | | British Columbia | | | |
| 1 | Boreal Forest N | CM11 | LBH11 | LBH14 | LBH16 | BWBSdk2 |  |  |  |
|  |  | NM11 |  |  |  |  |  |  |  |
|  |  |  |  |  |  |  |  |  |  |
| 2 | Boreal Forest Central | AP11 | CM12 | CM13 | DM11 | BWBSdk1 | BWBSmw1 | BWBSmw2 | BWBSwk2 |
|  |  | DM12 | DM13 | LBH13 | LBH15 |  |  |  |  |
|  |  | PAD11 | UBH13 |  |  |  |  |  |  |
|  |  |  |  |  |  |  |  |  |  |
| 3 | Lower Foothills | CM21 | CM32 | CM33 | CM34 |  |  |  |  |
|  |  | DM23 | LF12 | LF13 | LF15 |  |  |  |  |
|  |  | LF22 |  |  |  |  |  |  |  |
|  |  |  |  |  |  |  |  |  |  |
| 4 | Boreal Forest S | CM22 | CM23 | CM24 | CM31 |  |  |  |  |
|  |  | DM21 | DM22 | LF11 | LF14 |  |  |  |  |
|  |  | LF21 | PRP11 |  |  |  |  |  |  |
|  |  |  |  |  |  |  |  |  |  |
| 5 | Montane, Upper Foothills | M11 | M22 | M44 | M54 |  |  |  |  |
|  |  | M55 | M56 | SA11 | SA22 |  |  |  |  |
|  |  | SA31 | SA32 | UF11 | UF13 |  |  |  |  |
|  |  | UF24 | UF25 |  |  |  |  |  |  |
|  |  |  |  |  |  |  |  |  |  |
| 6 | Sub-boreal |  |  |  |  | SBPSxc | SBSmc2 | SBSmk1 | SBSmm |
|  |  |  |  |  |  | SBSwk2 | SBSwk3 |  |  |
|  |  |  |  |  |  |  |  |  |  |
| 7 | Sub-boreal |  |  |  |  | SBPSmk | SBSdk | SBSdw1 | SBSdw2 |
|  |  |  |  |  |  | SBSdw3 | SBSmc3 | SBSmk2 | SBSmw |
|  |  |  |  |  |  | SBSvk | SBSwk1 |  |  |
|  |  |  |  |  |  |  |  |  |  |
| 8 | Interior Mountains N |  |  |  |  | ESSFdc2 | ESSFdk | ESSFmv4 | ESSFmw |
|  |  |  |  |  |  | ESSFwc3 | ESSFwc4 | ESSFwm | ESSFxc |
|  |  |  |  |  |  | MSdm2 | MSxk | MSxv |  |
|  |  |  |  |  |  |  |  |  |  |
| 9 | Interior Mountains S |  |  |  |  | ESSFdc1 | ESSFdv | ESSFmc | ESSFmv3 |
|  |  |  |  |  |  | ESSFwc1 | ESSFwc2 | ESSFwk1 | MSdc2 |
|  |  |  |  |  |  | MSdk | MSdm1 | MSun |  |
|  |  |  |  |  |  |  |  |  |  |
| 10 | Interior Valleys SW |  |  |  |  | ICHdk | ICHmc2 | ICHmk3 | ICHmm |
|  |  |  |  |  |  | ICHmw2 | ICHvk1 | ICHwk1 | ICHwk2 |
|  |  |  |  |  |  | ICHwk4 | IDFdk1 |  |  |
|  |  |  |  |  |  |  |  |  |  |
| 11 | Interior Valleys S |  |  |  |  | ICHdw | ICHmk1 | ICHmw1 | ICHmw3 |
|  |  |  |  |  |  | IDFdk2 | IDFdm1 | IDFdm2 | IDFmw2 |

*The numbers and names of the groups correspond to Fig. 3c.

‡ Capital letters in the variant abbreviations represent the second level of Alberta’s Natural Regions classification and the ecological zones of British Columbia’s Biogeoclimatic Ecosystem Classification: Natural Subregions Alberta: A, Alpine; SA, Subalpine; M Montane; CM, Central Mixedwood; DM, Dry Mixedwood; NM, Northern Mixedwood; BSA, Boreal Subarctic; PAD, Peace-Athabasca Delta; LBH, Lower Boreal Highlands; UBH, Upper Boreal Highlands; AP, Athabasca Plain; LF, Lower Foothills; UF, Upper Foothills; FP, Foothills Parkland; PRP, Peace River Parkland. Ecological zones British Columbia: BWBS, Boreal White and Black Spruce; SBPS, Sub-Boreal Pine–Spruce; SBS, Sub-Boreal Spruce; ESSF, Engelmann Spruce–Subalpine Fir; MS, Montane Spruce; IDF, Interior Douglas-fir; CDF, Coastal Douglas-fir; ICH, Interior Cedar-Hemlock; CWH, Coastal Western Hemlock.

Table S7. Groups of similarly adapted seedlots of lodgepole pine, derived by the multivariate regression tree analysis based on ecosystem variants, corresponding to the groups in Fig. 4c

|  |  | Ecovariants by province‡ | | | | | | | |
| --- | --- | --- | --- | --- | --- | --- | --- | --- | --- |
| Group* | Group name* | Alberta | | | | British Columbia | | | |
| 1 | Montane AB | A13 | BSA11 | BSA12 | FP11 | BWBSdk1 | BWBSdk2 |  |  |
|  |  | FP12 | LF21 | LF22 | LF23 |  |  |  |  |
|  |  | SA11 | SA12 | SA22 | SA31 |  |  |  |  |
|  |  | SA32 | SA41 | SA42 | UBH12 |  |  |  |  |
|  |  | UBH13 | UF11 | UF12 | UF13 |  |  |  |  |
|  |  | UF14 | UF15 |  |  |  |  |  |  |
|  |  |  |  |  |  |  |  |  |  |
| 2 | Lower Foothills, NE of BC | LF11 | LF12 | LF13 | LF14 | BWBSmw1 | BWBSmw2 | BWBSwk2 |  |
|  |  | LF15 | UF24 | UF25 |  |  |  |  |  |
|  |  |  |  |  |  |  |  |  |  |
| 3 | Montane BC | LBH14 | M55 | M56 |  | ESSFdc1 | ESSFdk | ESSFmv1 | ESSFwc2 |
|  |  |  |  |  |  | ESSFwc4 | ESSFwm | ESSFxc | ESSFxv2 |
|  |  |  |  |  |  |  |  |  |  |
| 4 | Lower Boreal Highlands AB | LBH11 | LBH16 | M11 | M21 | ESSFdc2 | ESSFdv | ESSFmm1 | ESSFmv3 |
|  |  | M54 |  |  |  | ESSFmv4 | ESSFwk1 | ESSFwv |  |
|  |  |  |  |  |  |  |  |  |  |
| 5 | Dry Mixedwood AB | CM33 | CM34 | DM12 | DM13 |  |  |  |  |
|  |  |  |  |  |  |  |  |  |  |
|  |  |  |  |  |  |  |  |  |  |
| 6 | Sub-boreal |  |  |  |  | ICHvk1 | ICHwc | ICHwk4 | IDFxh1 |
|  |  |  |  |  |  | IDFxh2 | MSdc1 | MSdm1 | MSdm2 |
|  |  |  |  |  |  | MSun | MSxk | MSxv | SBPSmc |
|  |  |  |  |  |  | SBPSxc | SBSdk | SBSmk1 | SBSmk2 |
|  |  |  |  |  |  | SBSmm | SBSun | SBSwk1 | SBSwk2 |
|  |  |  |  |  |  | SBSwk3 |  |  |  |
|  |  |  |  |  |  |  |  |  |  |
| 7 | Sub-boreal |  |  |  |  | ICHmk1 | IDFdk1 | IDFdk2 | IDFdk3 |
|  |  |  |  |  |  | IDFdk4 | MSdk | SBPSdc | SBPSmk |
|  |  |  |  |  |  | SBSdw1/mw | SBSdw2 | SBSdw3 | SBSmc1 |
|  |  |  |  |  |  | SBSmc2 | SBSmc3 |  |  |
|  |  |  |  |  |  |  |  |  |  |
| 8 | Interior Valleys |  |  |  |  | ICHdw | ICHmc2 | ICHmw1 | ICHmw2 |
|  |  |  |  |  |  | ICHmw3 | IDFdm1 | IDFdm2 | IDFmw1 |
|  |  |  |  |  |  | IDFmw2 | SBSdh1 | SBSmh |  |
|  |  |  |  |  |  |  |  |  |  |
| 9 | Coastal BC |  |  |  |  | CDFmm | CWHds1 | CWHmm2 | CWHvm1 |
|  |  |  |  |  |  | CWHwh1 | CWHws1 | CWHxm1 | CWHxm2 |

*The numbers and names of the groups correspond to Fig. 4c.

‡Explanations for the abbreviations of the ecosystem variants are given in Table S6.
